# Context Modulates the ERP Signature of Contour Integration

**DOI:** 10.1371/journal.pone.0025151

**Published:** 2011-09-19

**Authors:** Bart Machilsen, Nikolay Novitskiy, Kathleen Vancleef, Johan Wagemans

**Affiliations:** Laboratory of Experimental Psychology, University of Leuven (K.U.Leuven), Leuven, Belgium; Ecole Polytechnique Federale de Lausanne, Switzerland

## Abstract

We investigated how the electrophysiological signature of contour integration is changed by the context in which a contour is embedded. Specifically, we manipulated the orientations of Gabor elements surrounding an embedded shape outline. The amplitudes of early visual components over posterior scalp regions were changed by the presence of a contour, and by the orientation of elements surrounding the contour. Differences in context type had an effect on the early P1 and N1 components, but not on the later P2 component. The presence of a contour had an effect on the N1 and P2 components, but not on the earlier P1 component. A modulatory effect of context on contour integration was observed on the N1 component. These results highlight the importance of the context in which contour integration takes place.

## Introduction

Our visual system provides us with a stable and coherent representation of the external world. An important intermediate step to achieve this stability is to determine which parts of the retinal input belong together, a process known as perceptual grouping. Vision takes advantage of statistical regularities in the input image to guide perceptual grouping processes. Adjacent elements of a shape outline are usually locally aligned. Detection of this collinearity might hence serve as a cue to the presence of a contour [1].

The importance of collinearity as a cue for contour integration is illustrated in the *snake detection* or *pathfinder* paradigm (for a review, see [2]), in which participants have to detect a contour in a cluttered background. The strength of contour grouping depends on the spacing and orientations of elements relative to the path orientation [3], [4]. Similar principles apply in the multistable organization of regular arrays of elements in rows and columns [5],[6]. Other, more global stimulus properties, also seem to influence the binding of local contour elements: Closed contours are more readily detected than open ones [7], [8], and symmetric contours are easier to detect than asymmetric ones [9].

Grouping spatially separated elements into a global structure requires integration beyond the classical receptive field (RF) size of V1 neurons [10]. Animal physiology studies have suggested that lateral connections within primary visual cortex could underlie the modulatory effects of sensory stimulation in the near RF surround of a V1 cell (e.g., [11]). However, contextual modulation by stimuli far outside the RF (e.g., [12], [13]) probably requires additional feedback from extrastriate regions [14]. Moreover, even contextual modulation by stimuli in the near surround cannot always be explained in terms of lateral connections. For instance, perceptual grouping of nearby context elements into a coherent configuration greatly reduces the influence of these context elements on a vernier-offset discrimination task [15].

Contextual modulations, by elements in the near and far surround, suggest that the process of contour integration can also be influenced by the context in which the contour is embedded. Indeed, contour integration improves when elements in the near surround are oriented perpendicular to the contour, and deteriorates when these elements are oriented parallel to the contour [16]. Shape detection benefits from having iso-oriented elements in the interior of a collinear shape outline [17], and a familiar object is easier to identify in a field of iso-oriented Gabor elements than in a field of randomly-oriented elements [18].

In the present study we take advantage of the high temporal resolution of electroencephalography (EEG) to investigate how the neural correlates of contour integration are modulated by the specific context in which a contour is embedded. We measure EEG activity in response to displays with and without a contour, embedded in a context of iso-oriented or randomly-oriented elements.

## Methods

### Participants

Twelve neurologically healthy participants (aged 21–36 years, 7 women) took part in the study. They had normal or corrected-to-normal vision. All participants were naive to the purpose of the experiment. The authors confirm that the research has been conducted according to all ethical standards imposed by their Ethics Committee at the University of Leuven, who approved the study. Written informed consent was obtained by all participants, according to the procedures imposed and approved by the above Ethics Committee.

### Stimuli

**Figure 1:**
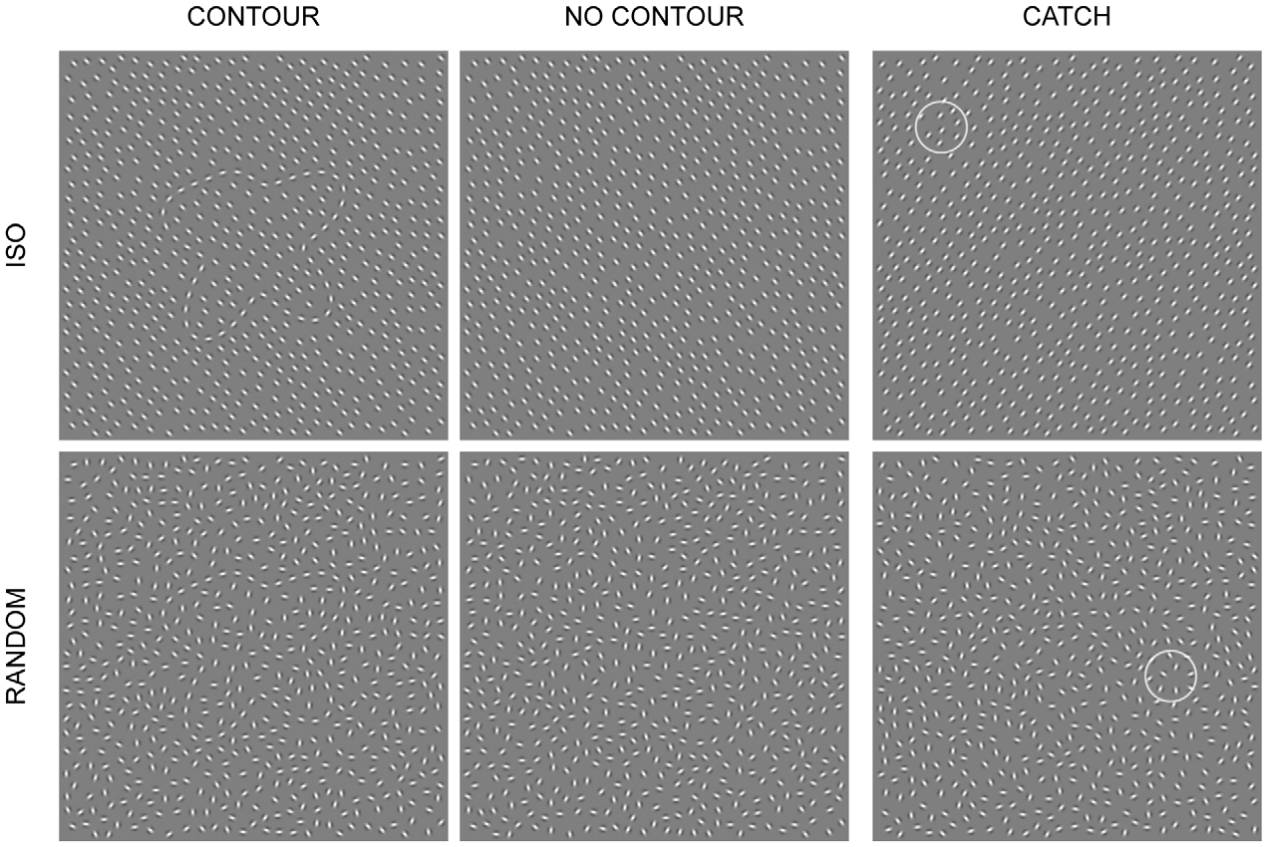
Example stimuli for the different array types. By rotating the elements of the contour condition (left column), the embedded contour perceptually disappears (middle column). Top row: Iso-oriented array, in which all elements surrounding the contour have the same orientation. Bottom row: Randomly-oriented array, in which the surrounding elements have a random orientation. Catch trials (right column) consist of the same stimuli with a small circle overlaid at a random location (here only illustrated for no-contour stimuli). Catch trials are not included in the analyses.

We used Matlab (v 7.1; The MathWorks, Natick, MA) and GERT, the Grouping Elements Rendering Toolbox (Demeyer & Machilsen, manuscript in preparation), to construct arrays of nonoverlapping Gabor elements on a grey background (Figure 1). The arrays comprised 496×496 pixels. Each Gabor element was defined as the product of a sinusoidal luminance grating (frequency of 4 cycles per degree of visual angle) and a circular Gaussian (standard deviation of 4 arcminutes). A subset of 45 Gabor elements was positioned along the contour outline of an artificial shape. The shape outlines were generated by plotting the sum of 5 radial frequency components (each sine wave having a random phase angle) in polar coordinates (see [9] for more details on similar stimulus construction). After rescaling the surface area to one eighth of the array size we colocalized the center of mass of each shape with the center of the array.

Next, the remainder of the array was populated with Gabor elements. To ensure a homogeneous spacing of Gabor elements throughout the array, we adjusted the number of elements inside and outside the shape outline separately for each shape. The number of interior elements ranged between 60 and 72, the number of exterior elements between 507 and 542. The number of Gabor elements inside, outside and on the shape outline was balanced across conditions. No stimuli were included for which the mean local density – here defined as the average Euclidean distance from each element to its five nearest neighbors – differed more than 1 arc min between interior, contour, and exterior elements.

For our 2×2 factorial design we created 4 different types of arrays (Figure 1). They only differed in the orientations of Gabor elements. In *no-contour/iso-oriented* arrays all Gabor elements had the same orientation. In *no-contour/randomly-oriented* arrays all Gabor elements were oriented randomly. By orienting the 45 Gabor elements on the shape outline parallel to their local tangent a closed contour was visible in an otherwise homogeneous field of iso-oriented (*contour/iso-oriented* array) or randomly-oriented (*contour/randomly-oriented* array) Gabors.

### Procedure

In a passive-viewing task we measured spontaneous neural activity evoked by the presentation of a Gabor array. To ensure that participants' attention was kept to the displays we introduced an orthogonal catch task: While fixating in the middle of the screen, participants were asked to press the mouse button when a circle was present in the array (Figure 1). To avoid that participants would only focus on a small region of the display we randomized the position of the circles across catch trials.

Stimuli were presented in 4 blocks of about 5 minutes, with 2 blocks of iso-oriented Gabor elements and 2 blocks of randomly-oriented elements. The order of blocks was counterbalanced across participants. The stimulus order within a block was pseudo-randomized. A contour stimulus (frequency = 0.28) was always preceded by 1–5 no-contour stimuli with identical Gabor positions (frequency = 0.61).

A contour stimulus was always followed by a no-contour stimulus with different Gabor positions. To ensure that all effects pertain only to differences in element orientation (and not to differences in element position), the first no-contour stimulus following a contour stimulus was not included in the analyses. The positions of Gabor elements in successive no-contour arrays did not change, while their orientations did (each element was rotated at least 25 degrees away from its previous orientation). Catch trials (frequency = 0.11) were randomly intermixed in the stimulus sequence. Catch trials were not included in the analyses.

Each stimulus was presented for 106 ms. The duration of the inter-stimulus interval (ISI) was uniformly sampled between 800 and 1200 ms. A central fixation cross was shown during the ISI. The experiment was run using the Presentation software (Version 14.5, www.neurobs.com) on a DELL 17″ CRT monitor with a spatial resolution of 1024×768 pixels and a temporal resolution of 85 Hz. At a viewing distance of 70 cm our stimuli subtended approximately 14 degrees of visual angle. The sound-attenuated room was dimly lit during the experiment.

### Electrophysiology

#### EEG Recording

EEG was recorded using a Quik-Cap (Compumedics Neuroscan) embedded with 64 sintered electrodes, positioned conform to the 10-5 system [19]. The signal ground was recorded just posterior to electrode FPz. The reference electrode was located between electrodes Cz and CPz. The EEG signal was amplified with a SynAmps2 amplifier (digitization: 1000 Hz; analog bandpass filter: 0.1–40 Hz). Impedance was kept below 10 kOhm. Vertical and horizontal electrooculograms were recorded to monitor eye movements and blinks.

#### Offline Preprocessing

The EEG signal was re-referenced to the average of the two mastoids and lowpass filtered at 30 Hz. Second-order blind source identification (SOBI) was used to eliminate the effect of eye blinks on all electrodes [20]. Next, we epoched the continuous signal with an 800 ms time-window, starting 200 ms prior to stimulus onset. The average amplitude in this 200 ms interval was used for baseline correction. Epochs containing voltages exceeding ±100 µV in any channel were not included in further analyses (4.5 percent). To avoid motor-related artifacts we also excluded catch trials and trials in which the participant erroneously pressed the mouse button (false alarms, 0.06 percent). Robust averaging of the artifact free epochs yielded 4 condition-specific event-related potentials (ERPs) for each participant. Condition-specific grand-mean ERPs were obtained by averaging the individual ERPs. All offline preprocessing was done using the SPM8 toolbox (http://www.fil.ion.ucl.ac.uk/spm).

#### Statistical Analyses

First, we applied a cluster-based permutation test for multi-sensor analysis to select the electrodes that differentially respond to contour versus no-contour arrays (pooled over the iso-oriented and the randomly-oriented conditions). This technique corrects for multiple comparisons by clustering the data based on their spatial and temporal adjacency [21]. It is implemented in the Fieldtrip toolbox for EEG/MEG-analysis (Donders Institute for Brain, Cognition and Behaviour, Radboud University Nijmegen, the Netherlands. See http://www.ru.nl/neuroimaging/fieldtrip).

For each participant we then averaged the condition-specific ERPs over the selected channels of interest. Next, we performed a mean amplitude and a peak latency analysis on the resulting ERPs, focusing on 3 early visual components: P1 (60–140 ms), N1 (120–220 ms), and P2 (180–280 ms). Peak latencies and mean amplitudes were computed automatically for the three components. The peak latency was defined as the latency of the largest peak within each time-window. A peak latency analysis requires the same number of trials across participants and conditions. Across participants the minimum number of artifact free epochs per condition was 99. Epochs were removed at random to obtain an equal number of epochs for each condition and participant. Analyses of mean amplitudes do not require the same number of trials and were computed from the complete set of trials.

For each component we then performed a 2×2 repeated-measures ANOVA separately for amplitudes and latencies (SAS procedure MIXED, SAS version 9.2), using a robust sandwich estimator to compute the variance-covariance matrix of the fixed-effects parameters [22]. To account for variation between participants we included a random intercept for each subject.

## Results

### Electrodes of Interest

**Figure 2:**
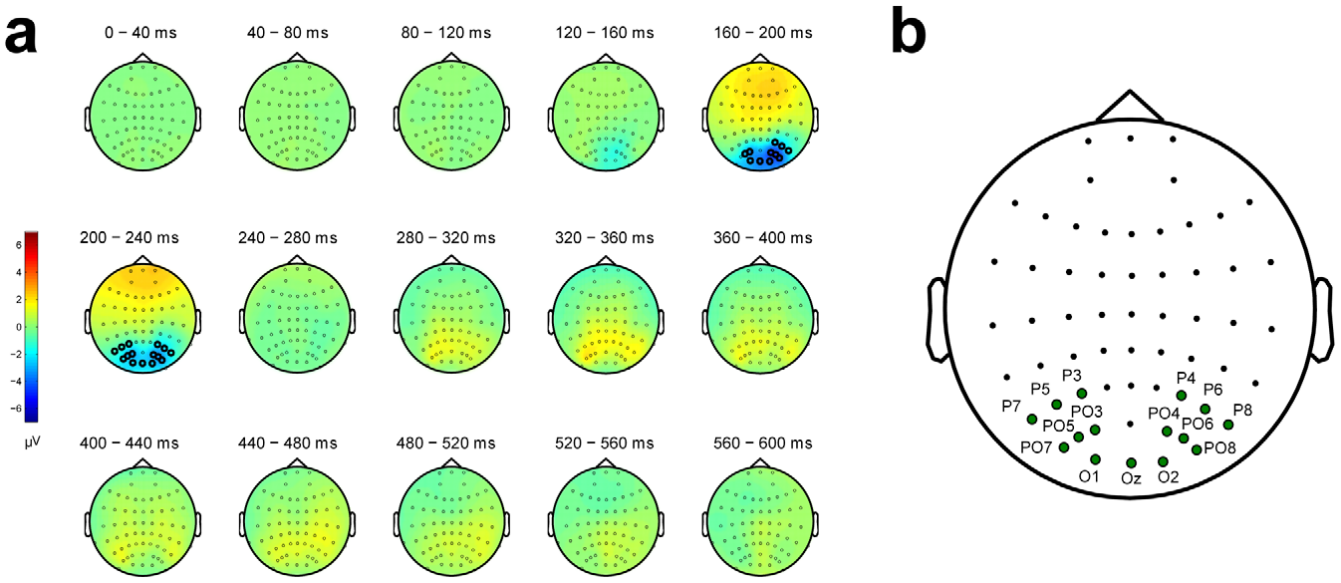
Electrodes of interest. (a) Topographic maps representing the temporal evolution of the difference between the contour and the no-contour conditions (aggregated over iso-oriented and randomly-oriented displays). The electrodes in the significant cluster are highlighted. (b) Spatial layout of the 15 electrodes of interest, with their corresponding labels.

The cluster-based permutation test found only one significant channel-time cluster (Monte Carlo *p*-value<0.001), consisting of 15 posterior electrodes (Oz, O1, O2, PO3, PO4, PO5, PO6, PO7, PO8, P3, P4, P5, P6, P7, P8). In the interval between 145 ms and 250 ms this cluster responded differently to displays with and displays without an embedded contour. Figure 2a shows the temporal evolution in topography for the difference between the average EEG in the contour and no-contour conditions. Significant electrodes are highlighted on the topographic maps. The ensemble of all electrodes belonging to the significant cluster is displayed in Figure 2b. The time-window and posterior location of this contour effect is consistent with previous studies on perceptual grouping (see Discussion).

### Analysis

**Figure 3:**
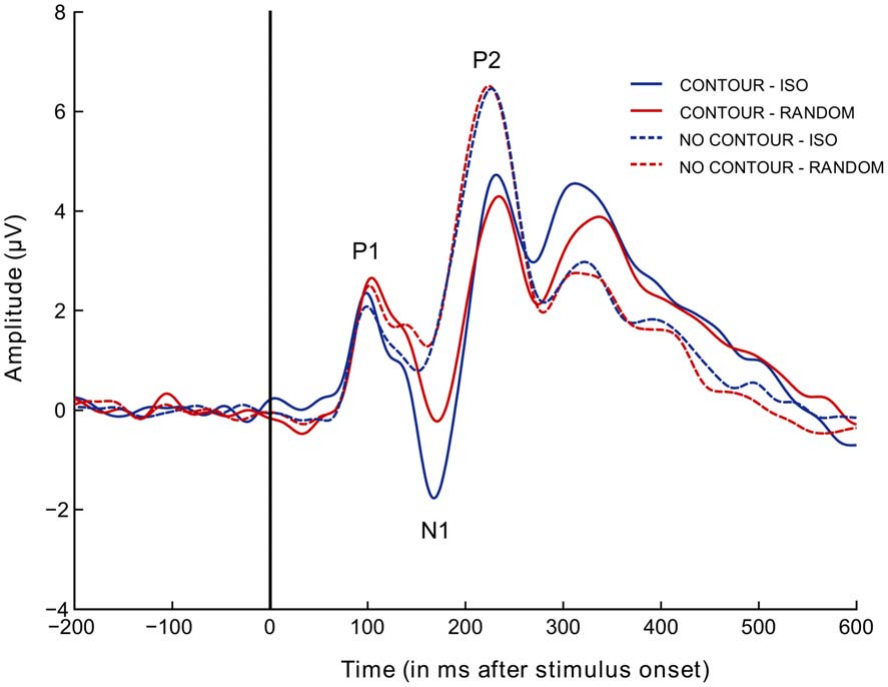
Condition-specific grand average ERPs. Grand mean ERPs (n = 12), averaged over the 15 electrodes of interest (Figure 2b). Full lines represent conditions with an embedded contour; dotted lines represent conditions without an embedded contour. Blue: Iso-oriented conditions. Red: Randomly-oriented conditions.

Figure 3 shows the condition-specific grand-mean ERPs. We will first describe the results of the analyses on the mean amplitudes for the 3 components of interest (P1, N1, and P2). Next, we describe the results for the complementary analyses on the peak latencies. The analyses are summarized in Table 1.

**Table 1:** Summary of the repeated-measures ANOVAs on the mean amplitudes and peak latencies for the P1, N1, and P2 components.

		Mean amplitude		Peak latency	
		*F*(1,33)	*p*	*F*(1,33)	*p*
**P1**	contour	0.76	0.3882	0.04	0.8493
	context	5.89	0.0208	2.20	0.1477
	contour×context	0.82	0.3725	0.74	0.3949
**N1**	contour	19.45	0.0001	10.27	0.0030
	context	8.70	0.0058	0.05	0.8163
	contour×context	5.75	0.0223	3.26	0.0802
**P2**	contour	33.93	<.0001	5.03	0.0318
	context	0.05	0.8178	0.00	0.9673
	contour×context	1.74	0.1958	0.96	0.3351

#### Mean Amplitudes

The mean amplitude of the P1 component was larger for randomly-oriented displays than for iso-oriented displays (*F*(1,33) = 5.89, *p* = 0.0208). The presence of a contour did not affect the amplitude of the P1 peak (*F*(1,33) = 0.76, *p* = 0.3882). The interaction between contour and context was also not significant (*F*(1,33) = 0.82, *p* = 0.3725).

We found main effects of contour (*F*(1,33) = 19.45, *p* = 0.0001) and context type (*F*(1,33) = 8.70, *p* = 0.0058) on the mean amplitude around the N1 peak. (Note that the main effect of contour was expected, as the selection of electrodes was based on the above cluster-based permutation test, contrasting conditions with and without an embedded contour. That analysis yielded one significant cluster in the interval between 145 and 250 ms, largely overlapping with the time-window of the N1 component). The interaction between contour and context was also significant (*F*(1,33) = 5.75, *p* = 0.0223): The effect of contour presence was larger for iso-oriented context than for randomly-oriented contexts. Note that when the same analysis was done on *peak* amplitudes instead of mean amplitudes, in the same time window but with an equal (and hence smaller) number of trials per condition (as for the peak latency analyses), this interaction effect did not reach significance (*F*(1,33) = 0.39; *p* = 0.54). A direct comparison between the two contour-present conditions was significant (*F*(1,33) = 5.32; *p* = 0.027).

Analyses of P2 mean amplitudes revealed a significant main effect for the presence of an embedded contour (*F*(1,33) = 33.93, *p*<0.0001). No effect of context type (*F*(1,33) = 0.05, *p* = 0.8178) was observed. The interaction between contour and context was also not significant (*F*(1,33) = 1.74, *p* = 0.1958).

#### Peak Latencies

The N1 component peaked later for displays with (162 ms) than for displays without (149 ms) an embedded contour (*F*(1,33) = 10.27, *p* = 0.0030). This effect was also present on the P2 peak (*F*(1,33) = 5.03, *p* = 0.0318): The P2 component reached its maximal amplitude on average 229 ms after stimulus onset for displays with an embedded contour, and 220 ms for displays without an embedded contour. The other analyses on peak latencies did not reveal significant effects (Table 1).

To summarize, we found that the presence of a contour increased the latency of the N1 and P2 components. We also found a main effect of context type on the mean amplitude of the P1 and N1 peaks, and a main effect of contour presence on the mean amplitude of the N1 and P2 peaks. The interaction between contour presence and context type was significant at the time of the N1 peak: The effect of contour presence was larger for contours embedded in iso-oriented backgrounds compared to contours embedded in randomly-oriented backgrounds. This modulatory effect of context on contour processing was no longer observed at the time of the P2 peak. Together, our results showed that the surrounding context of a contour modulated the neural signatures of contour grouping at the time of the N1 peak.

## Discussion

Our results demonstrate that a simple change in sensory input, i.e. in the orientations of elements surrounding an embedded contour, changes the neural correlates of contour integration. Early in the process of perceptual grouping, around the P1 peak (100 ms), iso-oriented and randomly-oriented backgrounds give rise to different ERPs, but no difference is observed for arrays with and without an embedded contour. The N1 occurs later and is larger when a contour is embedded in the array. It is also more pronounced for iso-oriented than for randomly-oriented displays. Importantly, the effect of contour presence is larger for iso-oriented than for randomly-oriented backgrounds. This points to a contextual modulation of contour integration around the time of the N1 peak (162 ms). The type of context no longer influences the ERPs at the time of the P2 peak (229 ms). The P2 peak only differentiates between displays with and without an embedded contour.

**Figure 4:**
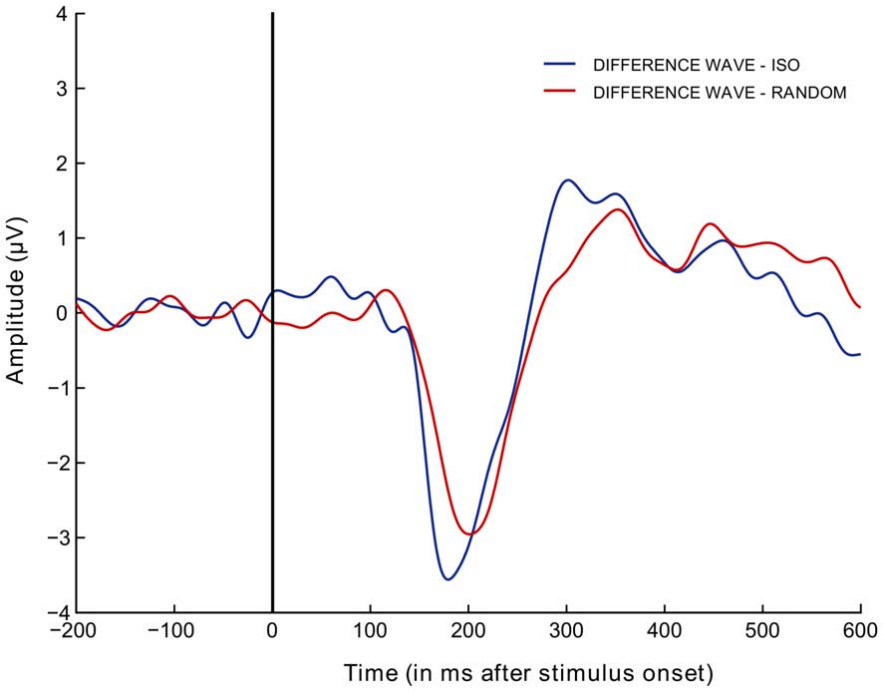
Difference waves for contour minus no-contour conditions. The graphs are obtained by subtracting the no-contour conditions (dotted lines in Figure 3) from the contour conditions (full lines in Figure 3).

The earliest visual evoked component is the C1, with a typical onset latency between 40 and 70 ms. Nonetheless, we did not include this component in our analyses. The C1 component changes polarity for stimuli presented above or below the horizontal meridian of the visual field, and is greatly reduced for stimuli covering the entire visual field [23]. The first component considered in our study is the P1 (60–140 ms) which does not show the polarity reversal for upper versus lower field stimulation. The P1 is generally assumed to reflect low-level physical stimulus attributes [24]. Our analysis on mean amplitudes is in line with this view: The P1 is modulated by the orientations of the context elements, but not by the presence of a global contour. A difference between contour and no-contour conditions only emerged after 130 ms, with a peak at 192 ms (mean latency of difference peak) after stimulus onset (Figure 4). The timing of this effect (reflected by the cluster-based analysis, and by the analyses on N1 and P2 amplitudes) is in good agreement with previous MEG and EEG studies on contour integration.

Tanskanen et al. [25] found that responses to collinear contours embedded in a field of randomly-oriented Gabors began to differ from no-contour stimuli after 130 ms, with the largest difference occurring 215 ms after stimulus onset. Earlier responses, 95 ms after stimulus onset, were identical to contour and no-contour stimuli. Their source analysis revealed contour-sensitive regions in the posterior parieto-occipital cortex. Mathes, Trenner and Fahle [26] found a contour-specific response, starting about 150 ms after stimulus onset, with detectable contours eliciting a negative shift over occipital recording sites. More demanding contour integration delayed and reduced the effect. Mathes and Fahle [27] used more difficult contours and observed a similar negativity at 220 ms after stimulus onset. In our study, the iso-oriented contexts produced an earlier and more pronounced negativity than the randomly-oriented contexts (Figure 4). This suggests that contour integration was probably easier in the iso-oriented displays (Figure 1). Although we did not measure detectability in the present study, an advantage for iso-oriented over randomly-oriented surrounds has previously been found in a figure-identification task [18].

Mathes et al. [26] have argued that the negative shift over posterior electrode sites is a fundamental feature of contour integration (see also [27]), and have related this finding to a well-known texture-segregation potential (tsVEP). The resemblance is indeed striking. The tsVEP is also indexed by a negative amplitude shift over posterior electrodes in a comparable time-window [28]. Mathes et al. [26] assume that a common border-detection mechanism accounts for the similarity between the tsVEP and the contour-specific negativity.

The negativity observed in contour-integration and texture-segmentation studies has also been reported in other EEG studies on perceptual grouping, in a comparable time window and with a similar topography (*similarity grouping*: [29]; *symmetry detection*: [30]; *circular Glass patterns*: [31]; *illusory contours*: [32]). It is therefore tempting to think of this early negativity as a general correlate of perceptual grouping processes. More research is needed to estimate the contribution of different perceptual processes to this posterior negativity.

The present study does not allow us to fully disentangle processes related to contour integration from additional processes involved in perceptual organization. The integration of elements into a perceptually closed contour invokes processes of figure-ground segregation, resulting in a specific depth ordering relationship between two regions: The area inside the contour is perceived as a figural region on top of a homogeneous (iso-oriented or randomly-oriented) background. Caputo and Casco [33] observed two negative peaks in the difference wave to uniform textures versus texture segregation displays at electrode Oz. The first negative peak (latency 140–160 ms) was related to texture segmentation per se, whereas the second negative peak (latency 200–260 ms) was influenced by global figure-ground segregation. With our experimental design we found only a single negative peak in the difference component (Figure 4). Interestingly, the temporal window of this peak comprised both peaks reported by Caputo and Casco [33]. It might be the case that the negativity in our difference wave does not only reflect contour-integration, but also processes related to figure-ground segregation.

A stimulus in which a figure is segregated from the background becomes perceptually more salient. Straube and Fahle ([34], see also [35]) investigated the effect of figure saliency on EEG responses. They found that perceptual saliency was inversely related to the amplitude of the posterior P2, regardless of the specific type of visual cue. Our results are in line with these findings. First, the P2 amplitude is smaller for contour stimuli than for no-contour stimuli. Second, the P2 component seems to reflect a cue-independent object representation, as its amplitude does not differ between iso-oriented and randomly-oriented contexts.

A clear figure-ground segregation also attracts participants' attention. Our orthogonal catch task was designed to ensure that attention was distributed uniformly across space throughout the experiment. However, this catch task does not preclude focused attention, i.e. the automatic deployment of attention to the figural region. In this regard differences in allocation of attentional resources might explain the changes in P2 amplitude, as already argued by Straube and Fahle [34].

Segregating the figural region from the background also invokes processes related to shape perception. Indeed, stimuli similar to ours have previously been used to study global shape perception (e.g., [36]).

Although the present study cannot dissociate between different components that contribute to the perceptual organization of the visual input, it is clear that in our stimuli integration of contour elements is a prerequisite for each of these processes. Figure-ground segregation, shape perception, figure saliency, and allocation of attention to the figural region all depend on the integration of contour elements (see also [37]). For this reason we decided to only focus on early visual components involved in contour integration.

By changing the orientations of all Gabors surrounding the contour we demonstrated the influence of context on the EEG response to collinear contour elements. This result does not imply that contour integration comes first and is then affected by the processes operating on the context elements. Instead, this result suggests an interplay between the processes involved in contour integration and the processes involved in figure-ground segregation. In this sense, integration and segregation are probably part of one process, a view which we have presented before [18], [37].

Our experimental design does not allow us to verify whether the contextual modulation is due to the global context, or to a spatially more restricted subset of Gabor elements close to the embedded contour. Dakin and Baruch [16] found a behavioral effect of local context by changing the orientations of elements (near-parallel versus near-orthogonal) in the immediate vicinity of a contour. They ascribe this modulatory effect to a surround-suppression mechanism and propose a tentative model of contour processing that explains the influence of local context. They also present some preliminary data showing that the strength of the contextual modulation increases asymptotically with the spatial extent of the context.

### Conclusion

Our study provides evidence that context modulates the electrophysiological signature of contour integration at early stages of visual processing. The effect of context was measurable at the time of the P1 peak. It was large at the time of the N1 peak, but absent at the time of the P2 peak. The effect of contour integration was not present at the time of the P1 peak. It only started around the N1 peak and was still present at the P2 peak. A modulatory effect of context on contour integration was observed at the time of the N1 peak. The presence of a contour had a more pronounced effect on the N1 peak when it was embedded in iso-oriented background elements compared to randomly-oriented background elements. These results highlight the dynamic interplay between perceptual grouping and the context in which it operates.
